# The Morphogenesis of Speech Gestures: From Local Computations to Global Patterns

**DOI:** 10.3389/fpsyg.2019.02395

**Published:** 2019-11-12

**Authors:** Khalil Iskarous

**Affiliations:** Department of Linguistics, University of Southern California, Los Angeles, CA, United States

**Keywords:** speech gestures, morphogenesis, BVAM system, Turing bifurcation, Hopf bifurcation

## Abstract

A subtle property of speech gestures is the fact that they are spatially and temporally extended, meaning that phonological contrasts are expressed using spatially extended *constrictions*, and have a finite duration. This paper shows how this spatiotemporal particulation of the vocal tract, for the purpose of linguistic signaling, comes about. It is argued that local uniform computations among topographically organized microscopic units that either constrict or relax individual points of the vocal tract yield the global spatiotemporal macroscopic structures we call constrictions, the locus of phonological contrast. The dynamical process is a morphogenetic one, based on the Turing and Hopf patterns of mathematical physics and biology. It is shown that reaction-diffusion equations, which are introduced in a tutorial mathematical style, with simultaneous Turing and Hopf patterns predict the spatiotemporal particulation, as well as concrete properties of speech gestures, namely the pivoting of constrictions, as well as the intermediate value of proportional time to peak velocity, which is well-studied and observed. The goal of the paper is to contribute to Bernstein’s program of understanding motor processes as the emergence of low degree of freedom descriptions from high degree of freedom systems by actually pointing to specific, predictive, dynamics that yield speech gestures from a reaction-diffusion morphogenetic process.

## Introduction

Languages vary widely in the phonological contrasts they utilize, and in the phonetic expression of these contrasts. However, two properties of the articulatory expression of phonological contrasts that occur universally are their particulation in time and space: articulatory gestures that physically express linguistic contrasts start at certain moments in time, and end at some later point in time, lasting some number of milliseconds (hence, their temporal particulation), and they are localized to some spatial *constriction* that extends for some number of millimeters (hence their spatial particulation). This is a subtle abstract property of contrastive gestures, which seems to not possibly be otherwise, as all motoric events have duration and if they are realized in space, they will be localized to a finite extent of space. It seems, therefore, that if phonological contrasts are spatiotemporally expressed, then, trivially and necessarily, they will be particulated. However, the neural system that organizes speech is known to be capable of very fast action (on the order of a few milliseconds), far faster than the durations of consonants and vowels (on the order of several to many tens or hundreds of milliseconds), and the motoneurons that control the speech articulators are spatially highly differentiated, capable of activity within a millimeter, but the constrictions of vowels and consonants are typically on the order of many millimeters or even centimeters (Zur et al., 2004; Mu and Sanders, 2010; Sanders et al., 2013). How and why then does the motor system organize gestures into spatiotemporal macroscopic units that are spatially and temporally large, compared to the neuromuscular fast and short-scale units?^1^ The fact of particulation, I believe, is evident to anyone who knows about phonetics and phonology, when it is pointed out, but its ubiquity, this work contends, is to be explained, as it is fundamental to our understanding of the interrelation of phonetics and phonology. This is because the linguistic pattering of the motor speech system makes use of gestural locality in space and time for signaling. Indeed, the particulation of contrastive units has been discussed explicitly or implicitly by several theories concerned with how the speech production and perception systems are able to fulfill their linguistic purpose. Some of these theories focus almost entirely on the articulatory system as the mechanism of particulation, while others focus on the acoustic-perceptual functioning of the vocal tract resonator. These theories will be presented in the next few paragraphs, and the reasons for the current model are advanced.

One explicit reference to the notion of particulation as fundamental to how language works through the articulatory system is the work of Studdert-Kennedy (Studdert-Kennedy, 1998; Studdert-Kennedy and Goldstein, 2003). Duality of patterning (Hockett, 1960) requires few meaningless elements to combine in many ways to create a very large number of meaningful elements (morphemes), and particulation in space and time is necessary for the generation of the large number of morphemes (Abler, 1989). That is, a large number of morphemes requires complex combinatoriality, and the latter requires particulation in space and time of the meaningless elements. This is indeed a very good reason for there to be particulation in time and space, and this paper does not contest this reason for the necessity of particulation, but this reasoning encapsulates a final cause (Aristotle, 1975), whereas the interest here is in the formal and material causes: how it is that the microscopic units of planning and execution are capable of realizing temporally and spatially macroscopic articulatory gestures? A material cause for particulation has been advanced by Studdert-Kennedy and Goldstein (2003) who attribute it to the anatomy of the vocal tract, where some articulators are separate from each other like the velum, tongue, and lips, so particulation is materially already present in the anatomy. However, the tongue cannot be claimed to be anatomically particulated into tip, dorsum, and root, since the muscles of the tongue, internal and external, interdigitate throughout the tongue (Sanders and Mu, 2013). The mystery of particulation, therefore, is not likely to be solved by positing separate organs of speech in the vocal tract, since the organ that probably contributes the most contrasts to the differentiation among speech sounds, the tongue, is not particulated anatomically. A formal cause of particulation has been proposed within the Task-Dynamic Model and Articulatory Phonology (Browman and Goldstein, 1989; Saltzman and Munhall, 1989; Sorenson and Gafos, 2016; Tilsen, 2019), which assume articulatory tasks to be expressed in terms of Constriction Locations and Degrees defined as finite spatial constrictions which control the vocal tract from specific initial to final points in time. So, particulation is built-in, and there is no problem to solve. The goal here will be to derive the spatial and temporal particulation, rather than stipulate it from the outset.

Another route to particulation is based on the acoustic-perceptual purpose of the vocal tract. Quantal theory (Stevens, 1972, 1989) motivated the typologically common vowels and consonants by considering the mapping between articulatory scales and the corresponding acoustic scale. If the articulatory scale is the position of a constriction in the vocal tract, and the corresponding acoustic scale is the position of the formants, then in many locations in the vocal tract, changing the position of the constriction slightly changes a few of the low formants very little, whereas in some locations, changing constriction location slightly changes a few of the low formants drastically. Stevens (1972, 1989) argued that languages choose locations in the vocal tract where constriction change has a small effect on the formants, as stability would make, for instance, coarticulatory and token-to-token variability in exact constriction positioning have less of an acoustic effect. A theory that motivates spatial particulation from Quantal Theory is distinctive regions and modes (Mrayati et al., 1988). The theory starts from the sensitivity functions showing how constrictions affect the first three formants, and show that there are eight discrete quantal regions, where a constriction in one of these regions has the same distinctive qualitative effect on the three formants. In one of these eight regions, for instance, F1, F2, and F3 are all raised, while in another, F2 and F3 are raised, but F1 is lowered. The regions are distinctive in that each of them has a different formant behavior. This cause for particulation, like that presented earlier based on the duality of patterning and the need for many words for successful communication, is a final/teleological cause. The point of this paper is that there are formal and material causes that make the related final causes of linguistic combinatoriality and discrete acoustic behavior possible.

It will be shown that particulation emerges from distributed local computations between many units expressing the neural networks of the central and/or peripheral regions responsible for the control of the vocal tract articulators, as well as the interaction of the points of the hydrostatic tissues that compose the tongue, velum, lips, and vocal folds. That is, if articulation is controlled by topographically organized interactive neural networks, whose units constrict and relax the vocal tract at specific points, then we predict the spatial extent of contrastive units to be finite in extent, and to extend temporally to finite durations. This particulation can then serve the purpose of combinatoriality and quantal region behavior. Moreover, the model to be presented does not just provide a high-level description, but rather makes very specific predictions about how constrictions evolve in time, which allows it to be tested against existing data.

The goal is not to replace the ideas of articulatory phonology, but to derive the notions of Task Constriction Location and Degree, as well as the finite temporal extent of gestures from primitive computational principles that we know govern the neural and muscular tissue. Instead of seeing the neural and muscular unit firings as the final actuator of a planning process that assumes particulation, I show that global particulated gestures emerge from a local pattern formation process. The focus of this paper will be on the aspects of speech that the tongue contributes to, but it is believed that the contribution of articulators can be accounted for in the future using similar techniques. Section “Turing and Hopf Particulation” describes the Reaction-Diffusion Model of Turing and Rashevsky (Rashevsky, 1940; Turing, 1952; Rosen, 1968) describing inter-unit interaction that yield particulation either in space or in time, separately, in a tutorial style. Section “Simultaneous Turing and Hopf Patterning” shows simulations of the BVAM Reaction-Diffusion Model (Barrio et al., 1999) and how the computations lead to *simultaneous* patterning or particulation in space and time, which can be interpreted as area function change in speech production. It is also shown that two signatures of natural constriction dynamics, pivoting (Iskarous, 2005) and large proportional time to peak velocity in articulatory gestures (Sorenson and Gafos, 2016), are predicted by the BVAM theory. Section “Discussion and Conclusions” includes a discussion of what has been achieved and what needs to be resolved in future work.

## Turing and Hopf Particulation

After founding computer science and artificial intelligence, inventing the computer, and breaking the Nazi code, Alan Turing turned to what he perceived as the fundamental question of biology: how does biological structure form? He knew that biological structures are highly spatially articulated, but that they emerge from structures that are basically uniform. How does uniformity give way to nonuniformity of structure? This is what he called the problem of *morphogenesis*, which he felt was well-exemplified by the featureless symmetric blastula that then changes to a highly structured embryo. Another example is the stripes on zebra skin. The notion of *gene* was already understood, and Turing knew that some genetically initiated process in individual cells leads to local within-cell expression or lack of expression of melatonin. But why do the cells within a dark stripe express melatonin, while the cells in the light colored areas do not? That is, what is the origin of the global particulation of the skin? Turing’s answer was that genetically controlled uniform local interactions between cells is what gives rise to the global pattern formation. Turing proved that if the microscopic cells perform reactions, to be described, and if substances he called morphogens diffuse between surrounding cells, then these purely local microscopic *uniform* reactions (i.e., the same reactions and diffusions occur throughout the skin), lead to a local computation that yields highly particulated, nonuniform macroscopic structures. Before introducing the reader to the nature of these computations, and why the genesis of form in this way is so surprising, I would like to suggest that Turing’s ideas are relevant for the current discussion of the spatial particulation of phonological gestures, because it will be suggested that the same dynamic that Turing used for biological pattern formation is at the basis of the particulation of vocal tract into parts that are macroscopically constricted (in analogy with the black strip on the zebra) and parts that are unconstricted (in analogy with the white background on the zebra skin) based on the computations of many microscopic neural and muscular units. I would also like to add that even though Turing’s work was entirely mathematical, with no biochemical proof of any sort, the last two decades of work in laboratories throughout the world have biochemically *proven* that many animal biological structures such as hair follicles, digit development, where the five fingers and toes on each hand and foot emerge from a uniform stump through the reaction and diffusion of particular proteins in known amounts to create fingers (in analogy to black stripes), on a background of interfinger notches (white background), cortical folds in the brain, tooth development, and many other systems (Cartwright, 2002; Meinhardt, 2012; Sheth et al., 2012; Cooper et al., 2018). Therefore, the reaction-diffusion computational paradigm definitely seems to be relevant to biology, and could potentially be relevant to the biological behaviors of language and speech.

**Figure 1:**
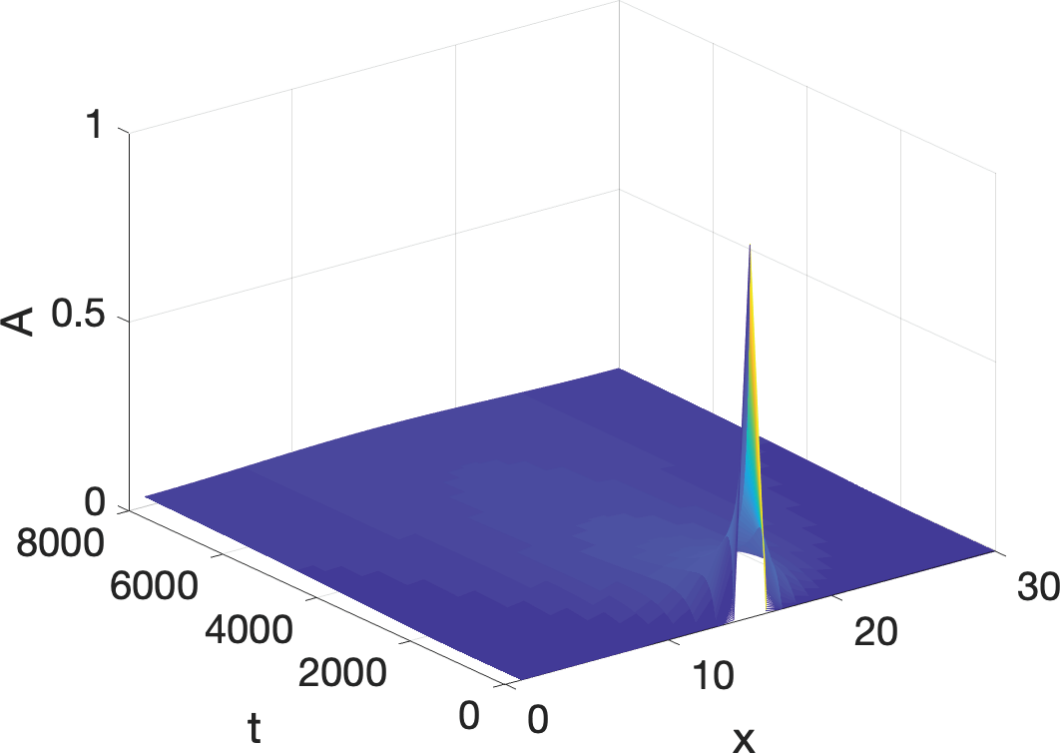
Diffusion as loss of structure and gain of uniformity.

Diffusion had been understood for many decades. To understand how diffusion works, imagine a sheet of units, with a variable *A* defined at each unit, and the value of *A* can change in time, and can be different at contiguous units. The dynamic of diffusion says that the value of *A*, at each step of time, should become more similar to the average value of *A* in the local surroundings of the unit. If this is the case, *diffusion* will be said to have occurred. Mathematically, this is expressed as:^2^

(1) $$\frac{\partial A}{\partial t}=D\nabla^{2}A\;$$

which says that the change of *A* with respect to time at each unit is directly proportional to the discrepancy between the values of *A* at the unit and the average value of *A* in the immediate surroundings (symbolized by ∇^2^ *A*, the Laplacian of *A*)^3^, and *D*, which indicates the diffusion coefficient. To get a feel for the latter factor, imagine ink diffusing in water vs. oil. Diffusion is much faster in water (*D* is high), and very slow in oil (*D* is very low). A graphical example of diffusion can be seen in Figure 1 in one dimension. At the initial step of time, the middle unit has *A* = 1, and all the other units are set to 0. At each subsequent frame in time, diffusion leads to less *A* where *A* had been high, since the surroundings have very low *A* (therefore their average is low), and *A* increases at points where the surrounding average is greater than their value. As time evolves contiguous units acquire closer values to the average of their surroundings, so that the initial nonuniformity of *A* = 1 in one location, and *A* = 0 at all other locations is replaced eventually becoming more and more uniform across the units.

What is easy to see is that diffusion erases structure, which is the way it had been understood for decades, and that is why it was surprising that Turing was able to prove that it was essential for morphogenesis in biology.

Even more surprising is that the other factor, reaction, also leads to uniformity. Imagine that at every point of a domain there are two substances, one we will call *A*, the Activator, and the other we will call *I*, the Inhibitor. The amounts of these substances are the values of the variables *A* and *I*. At each moment in time and at each location, if *A* is positive, then *A* increases with respect to time, so it is an Autoactivator. If *A* is positive, it also activates *I*, so *I* increases with respect to time. But when *I* is positive, *A* decreases. So *A* activates itself and *I*, but *I* inhibits *A*. This is therefore an Activator-Inhibitor reaction happening at each location. Mathematically, this reaction can be written as:

(2) $$\begin{array}{c} \frac{dA}{dt}=aA-bI\\ \frac{dI}{dt}=cA-dI \end{array}$$

**Figure 2:**
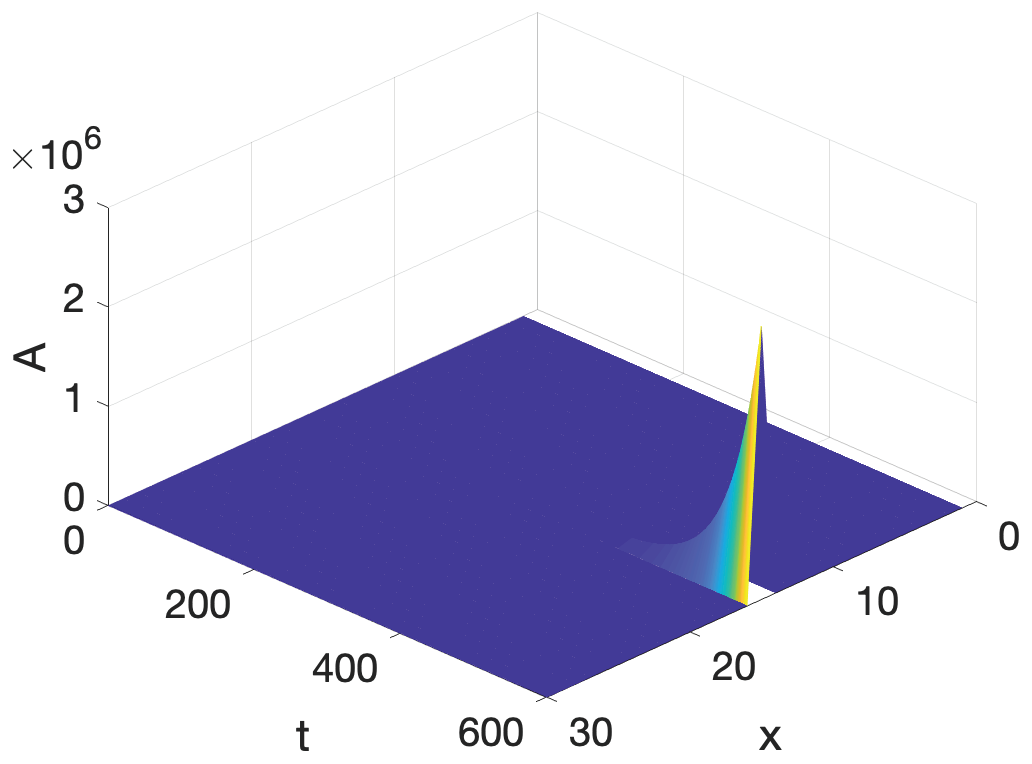
Activator-inhibitor reaction as loss of structure and gain of uniformity.

This means that *A* increases with respect to time if *A* is positive, and decreases if *I* is positive. The coefficients *a* and *b* indicate by how much the increases and decreases affect the increase/decrease in *A*. *I*, on the other hand, is increased with respect to time, if *A* is positive, but decreases, if it itself is positive (the last aspect is not essential). Figure 2 shows an example of the reaction in Equation 2, with *a* = *c* = 1, and *b* = *d* = 0.5. The initial conditions are the same as for the diffusion in Figure 1. Time is counted in frames, and the *A* variable, here and the rest of the paper, is in arbitrary units.

As we saw with diffusion, the activator-inhibitor reaction in this (the generic) situation leads to loss of structure present in the initial condition. How then, is it possible that an activator-inhibitor reaction combined with a diffusion, both of which lead to uniformity, usually called *equilibrium*, can lead to the birth rather than the death of nonuniformity and structure?

**Figure 3:**
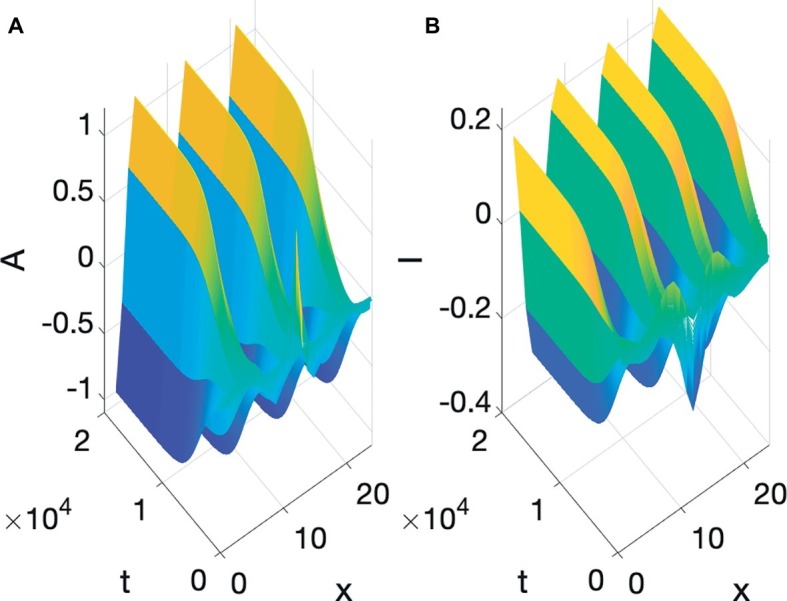
Turing pattern formation: **(A)** Activator, **(B)** Inhibitor.

Turing’s ingenious realization (Turing, 1952) was that if both *A* and *I* react in a way similar to Equation 2, and both diffuse, but *A* is slower to diffuse than *I*, i.e., *I* has a higher diffusion coefficient than *A*, then structure and particulation are born. The physical intuition is as follows: (1) if *A* is positive at a location, it autocatalyzes itself, and starts to diffuse slowly, also increasing *I*, which diffuses faster than *A*; (2) the fast diffusion of *I* leads to spots, outside of the region where *A* is most highly concentrated, where *I* is significant enough to inhibit *A* from spreading; therefore, *A* is confined into a macroscopic region of concentration. This region can be a stripe, a spot, finger, a hair, or a tooth. In one dimension a small random bump of *A* grows into a whole region where *A* is high and surrounding regions where *A* is negligible, but *I* is high, a situation we refer to as spatial particulation. An example of Turing pattern formation is shown in Figure 3, where the initial values are, again, all 0 except for a bump of *A* in the middle. Instead of decay to equilibrium, the bump, after suffering some decay, develops into a self-enhancing nonuniform, particulated pattern. Peaks of the inhibitor alternate with peaks of the activator. The reaction-diffusion equations simulated in Figure 3 will be discussed at length in the next Section.

Turing pattern formation describes the birth of global macroscopic nonuniformity from uniform local interactions of microscopic units. The fact that the interactions are uniform and local is quite significant: reaction involves locality in time, since the value of each point is affected by its immediate past and the past of the other substance, and diffusion involves locality in space, since the value of *A* and *I* are affected by the average of their local neighbors. The uniformity is that of the diffusion and reaction coefficients, which are the same everywhere. However, out of these uniform interactions, macroscopic regional differentiation, or pattern emerges.

**Figure 4:**
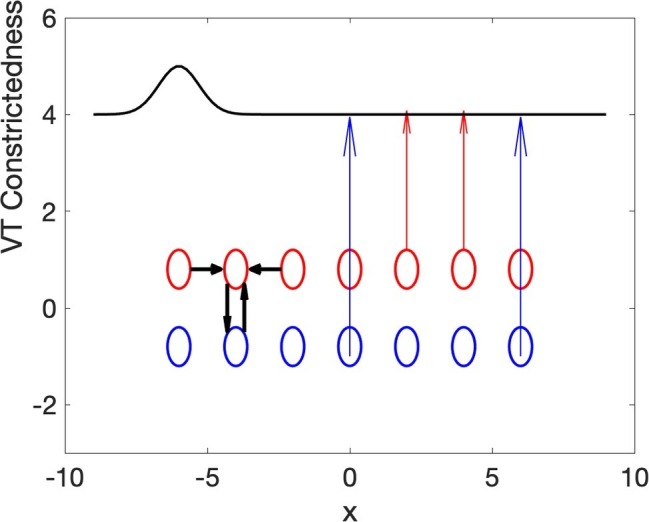
Model of constriction formation with a reaction-diffusion dynamic.

The idea advanced in this paper is that the spatial nonuniformity that emerges from local uniform Turing computations can also explain the spatial differentiation of the vocal tract into constricted and unconstricted areas. *A* in this case is interpreted as the constrictedness of the vocal tract. The computations involved here would involve many thousands of topographically organized neural units, where each unit controls the amount of opening at one point of the vocal tract (the area function). Evidence of topographic organization of parts of the brain that control tongue and lips is plentiful in the investigation of primates, including humans, on the cortical and subcortical levels (Waters et al., 1990; Choi et al., 2012; Kuehn et al., 2017). This is all relevant only if it is the case that neural computations can lead to a Turing pattern, but that this is possible was shown several decades ago by Ermentrout and Cowan (1979). In their model, the activator and inhibitor are the excitatory and inhibitory neurons well-known in neuroscience since the work of Sharrington (1932). The model assumed is depicted in Figure 4. The Excitatory/Activation units are in red, and the Inhibitory ones are in blue, and there could be many hundreds, if not thousands, of such units. Horizontal black arrows show the local diffusion connections for one neuron, and the vertical black connections show the local reaction connections for the same neuron. The units, as we know for units at different brain areas, are topographically organized, and each unit activates/constricts one point of the vocal tract, and the next neural unit activates the next in the vocal tract. Crucially, the vocal tract constriction is spatially macroscopic, compared to spatial extent of the microscopic units. Therefore, a single global, macroscopic constriction may be due to cooperative activity among hundreds, if not thousands, of tiny units interacting *locally* in space and time. The interactions, moreover, are assumed to be, so far, of the reaction-diffusion dynamic generating Turing patterns.

However, what the Turing pattern predicts is spatial differentiation, but the spatial pattern persists for all future time, and that is not, of course, what we see in speech, where constrictions are also delimited in time. In the rest of this section, we present how temporal particulation arises. A well-known way to particulate continuous time into periods of various durations was already known to Huygens in his invention of the pendulum clock. The linear nucleus of this model is what Meinhardt (1982) called an Activator-Substrate Model, but what is usually termed the Harmonic Oscillator seen in Equation 3:

(3) $$\begin{array}{c} \frac{dA}{dt}=bS\\ \frac{dS}{dt}=-cA \end{array}$$

**Figure 5:**
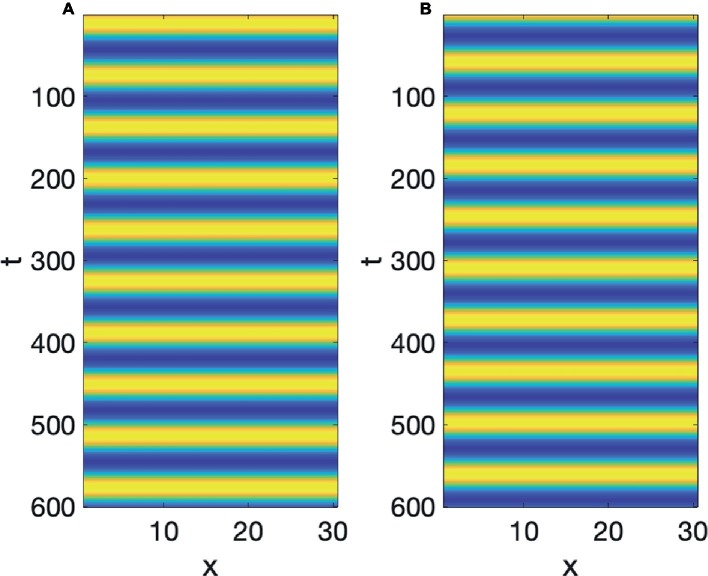
Linear oscillator with demarcation in time: **(A)** *A*, **(B)** *S*.

This is a reaction diffusion equation where *A* increases if *S* is positive, but when *A* grows positively, *S* decreases (this kind of reaction can also be the nucleus of a Turing pattern as shown by Meinhardt, 1982). Figures 5A,B show a simulation of the values of *A* and *S*, respectively, of Equation 3 with *b* = 4 and *c* = 1. We see that as time increases, *A* and *S* are demarcated in time, since the rise and fall, demarcating a period for each cycle of waning and growth, so continuous time has been demarcated into periods of equal length. It can also be seen that *A* and *S*, as they instantiate the constraints imposed by Equation 3, alternate in growth and decline, i.e., they are out-of-phase.

**Figure 6:**
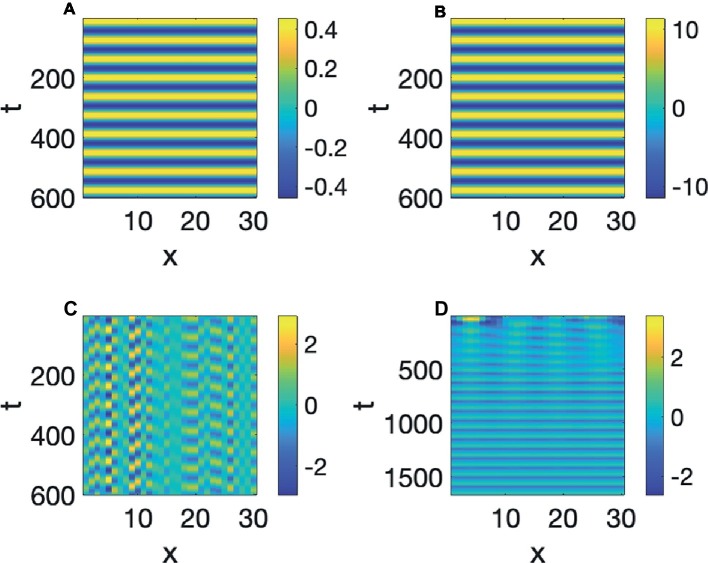
**(A–C)** Linear oscillators with initial amplitudes 0.5, 10, and random. **(D)** Hopf pattern.

In the simulation of Figure 5, both *A* and *S* were started with the value of 1 at the initial time. If the initial value of *A* and *S* were smaller, such as 0.5, then the value of *A* will oscillate up and down reaching that value positively and negatively as time progresses, and if the initial values were larger, such as 10, then that will also be the value of the extremes of *A*. Simulations of those two situations are in Figures 6A,B. We therefore say that the linear oscillator in Equation 3 is highly sensitive to the initial conditions, as its oscillatory amplitude is not stable, but varies with the initial amplitude. This can be seen clearly in Figure 6C, where the initial values are random. The oscillations at each point have different maximal amplitudes determined by the initial random values.

Huygens knew that a real clock needs to have oscillatory properties that are insensitive to the initial conditions, and the result was a more complex nonlinear version of Equation 3, which will be discussed in the next section, that will oscillate with a certain frequency and amplitude, regardless of how it initially started. Figure 6D shows an instance of such a Limit Cycle, or a Hopf Pattern, where the amplitude at the initial time is random. Regardless of the initial value, it can be seen that the oscillation develops to a stable value, demarcating time into equal increments, like a clock. These types of oscillations have been shown by Wiener and Rosenblueth (1946), Winfree (1980), and others to have extensive applications to biological systems. And some of the earliest evidence for Hopf patterns were to model neural oscillations (Wiener, 1958). Therefore, just as with Turing pattern formation, there is evidence that neural systems are capable of generating these patterns. Hopf patterns, have of course, also been made use of for understanding timing in speech production research (Byrd and Saltzman, 1998), but that work uses these oscillators to denote planning oscillators at a much higher mental level, whereas here, the *A* variable will be interpreted, literally to be the amplitude of opening of the vocal tract at specific locations x, in the vocal tract.

## Simultaneous Turing and Hopf Patterning

Speech production is built on particulation in both space and time, or as physicists would put it, necessitates the breaking of both spatial and temporal symmetry. The Turing and Hopf patterns seen in Figures 3, 5D can each be generated by a multitude of different differential equations (Cross and Hohenberg, 1993). However, it is very difficult to find equations, which simultaneously exhibit spatial and temporal patterning of the Turing and Hopf types,^4^ which is the situation necessary for modeling speech production, since we need both spatial and temporal demarcation. What we need are equations that admit of what are usually called Turing-Hopf interactions (Walgraef, 1997). However, many of the cases discussed in the literature under the banner of Turing-Hopf Bifurcations yields patterns too different from those we find in speech. An extensive search in the literature has yielded one set of reaction-diffusion equations that are a useful starting point, in the opinion of the author, for studying the kind of spatiotemporal particulation we find in speech. It is not expected, by any means that this is the only useful reaction-diffusion system that exhibits the patterning we need, but it is an interesting starting point. These equations are called Barrio-Varea-Aragon-Maini (BVAM) for their discoverers Barrio et al. (1999). They are listed in Equation 4:

(4) $$\begin{array}{c} \frac{du}{dt}=g\left(u+av-Cuv-uv^{2}\right)+D_{u}\nabla^{2}u\\ \frac{dv}{dt}=g\left(hu+bv+Cuv+uv^{2}\right)+D_{v}\nabla^{2}v \end{array}$$

**Figure 7:**
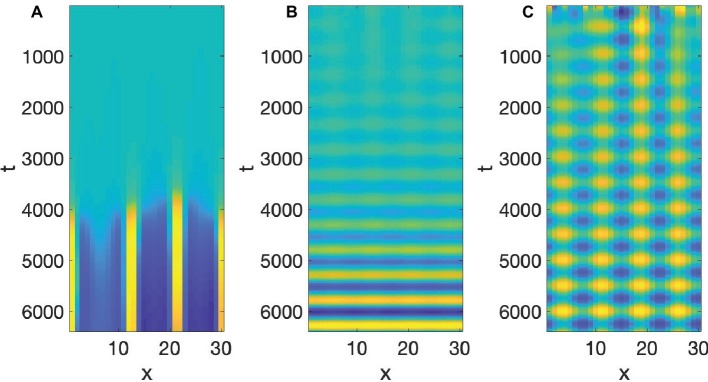
BVAM system **(A)** Turing, **(B)** Hopf, **(C)** Turing and Hopf patterns.

*u* and *v* are the activator and inhibitor variables, respectively, which interact in a nonlinear manner. The first two terms on the right-hand side are linear, the third term is quadratic, and the fourth is cubic. The signs of the cubic terms show that (from the first equation), when *v* is large positive, *u* will decrease, and (from the second equation) when *u* is large positive, *v* increases, confirming the activator-inhibitor nature of the reaction. The linear and quadratic terms in the equations, and the coefficients *a*, *b*, *C*, *g*, *h*, modulate the basic activator-inhibitor reaction. The last two terms in these equations are the diffusion terms, and the most important condition for a Turing pattern to emerge is that *D_v_* > *D_u_*. When we set *a* = 2.513, *C* = 2, *g* = 0.199, *h* = −1, *D_u_* = 0.122, and *D_v_* = 1, then the value of b will determine whether we get a Turing Pattern only (*b* = −1.95), Hopf Pattern only (*b* = −0.85), or simultaneous Turing and Hopf patterns (*b* = −0.95). The analysis showing the influence of *b* is due to Leppänen et al. (2003). Simulations of Equation 4 with the values just discussed can be seen in Figure 7.

To re-iterate, the simultaneous presence of both Turing and Hopf is very rare (non-generic or structurally unstable in the mathematical senses), and is due to the exact value of *b*. If that value is changed slightly, either a Hopf only or a Turing only pattern is obtained. What we have in Figure 7C, is therefore a very special situation, in which local interactions in space and time among many microscopic units yields a global pattern where space and time are demarcated in spatial and temporal intervals. However, all three of the patterns in Figure 7 are quite stable, as can be seen from the fact that the initial values of the simulations are random, but they reach stable patterns. The claim here is that the relevance of this to speech is that it is quite possible that the planning of the motion of points in the vocal tract, if it is done *via* reaction-diffusion type local uniform computations, could yield the type of particulation we find in speech production, if excited neural units seek to constrict the vocal tract and inhibitory neural units seek to open the vocal tract.

Two pieces of evidence for the relevance of simultaneous occurrence of Turing and Hopf pattern to speech is that the dynamics of constrictions, not just their presence, seems to be reproduced by the dynamic in Equation 4. Empirical studies of tongue motion in English and French by Iskarous (2005) showed that constrictions in speech are formed and relaxed in the same location, a pattern termed *pivoting*. In the production of [ia] for instance, the constriction dynamic for the [i] constriction relaxes within the palatal region, while the [a] constriction forms in the pharyngeal region. It was shown that there is very little change in the area function elsewhere in the vocal tract, including the areas between the palatal and pharyngeal regions. It may seem that this is trivial, and could not be otherwise, but we could imagine the palatal constriction to travel as a traveling wave down the vocal tract, fully formed, to the pharyngeal region. And indeed, the tongue is capable of generating such motion, since during swallowing a traveling wave of muscular activation pushes the bolus down the vocal tract with a constriction-like pusher of the bolus. However, investigation of hundreds of transitions between different speech segments showed that actual constriction formation (Iskarous, 2005) is more like a standing wave pattern of wave motion, where the formation and relaxation of constrictions occurs in the same place. And that is indeed the pattern we see in Figure 7C. The constriction peaks and troughs do not travel, but form and relax in the same location. Iskarous et al. (2010) have shown that the pivot dynamic plays a role in the perceptual system’s judgment of the naturalness of speech.

**Figure 8:**
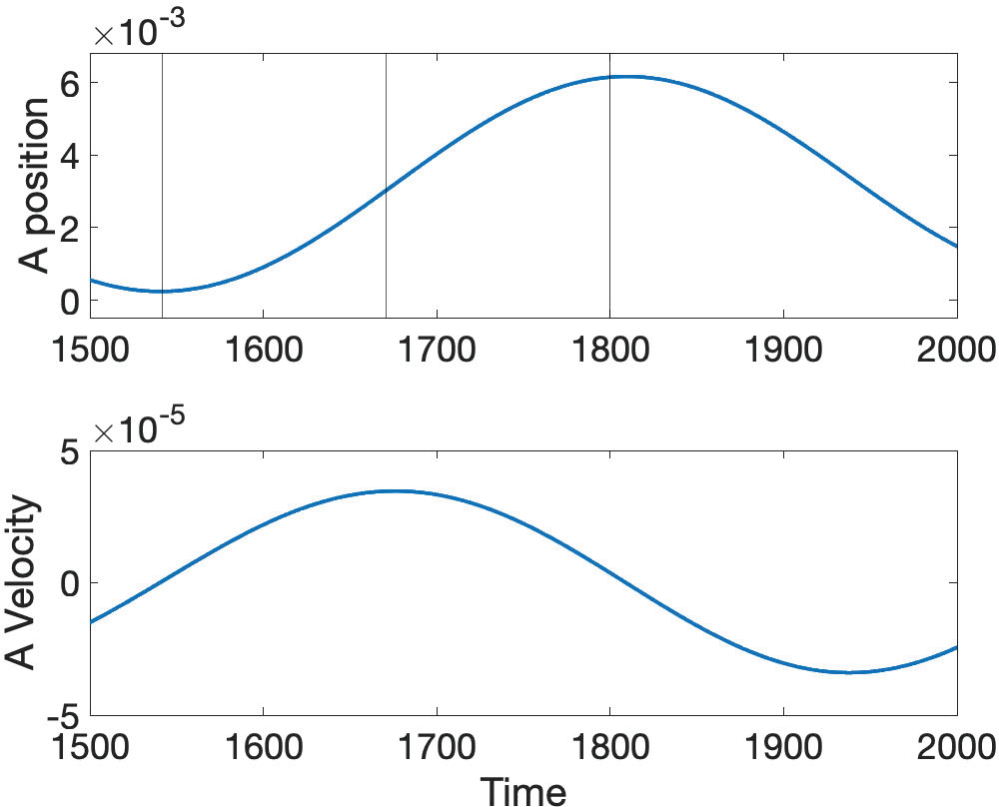
Position and velocity of *A* predicted by the simultaneous Turing and Hopf pattern of Equation 4.

Another well-studied aspect of speech dynamics is how constriction degree varies as time progresses. The initial hypothesis of Fowler et al. (1980) and Saltzman and Munhall (1989) was that the gestural dynamic is linear second order critically damped, but Perrier et al. (1988) and Sorenson and Gafos (2016) showed that the peak velocity in actual speech movements occurs about half way in the interval from lowest position amplitude to target position achievement, whereas the critically damped second order system predicts a much earlier proportional time to peak velocity (0.2). Figure 8 shows the position and velocity of *A* predicted through simulation of Equation 4. Proportional time to peak velocity is 0.49, which is close to the value observed and predicted by a cubic nonlinear dynamic in Sorenson and Gafos (2016).

The reason that the model is able to predict the late velocity peak is that the reaction dynamics in the Reaction-Diffusion model in Equation 4 is nonlinear, as in the model proposed by Sorenson and Gafos (2016). What have we gained through the proposed model then, if one can already predict the late peak velocity through that earlier model? The Sorenson and Gafos model, like the Saltzman and Munhall Task Dynamic Model are for point dynamics and by default is supposed to apply to all vocal tract gestures. Particulation in space in these models is postulated due to the inherent particulation of point dynamics. The proposed model is for the entire area function, and predicts particulation in space rather than stipulates it. One can of course say that the current model stipulates particulation through the specific constants and dynamics in Equation 4. However, Reaction-Diffusion dynamics can lead to equilibrium solutions, so particulation is a possible feature of solutions, but is not a necessary one, whereas when a point-dynamic is chosen, particulation in space is not only possible, but necessary. Furthermore, a prediction of the BVAM model, with the chosen coefficients, is that Constriction Degree and Constriction Location have entirely different dynamics, with Constriction Degree being reached gradually with a late peak velocity, as just discussed, but that Constriction Location changes using a pivoting dynamic, shifting discretely from one location to another, as in the earlier discussion on pivoting. This prediction is not shared with earlier models, which have no reason to predict Constriction Location and Degree to differ in their dynamics. In the current model location and degree occupy ontologically disparate parts of the mathematical architecture of the model. Constriction Location refers to a setting of the *independent variable* of position that happens to have a large amplitude due to Turing and Hopf pattern formation, whereas Constriction Degree refers to the large amplitude itself, not its location. The difference in dynamics is due to the ontological difference. We do not take this work to be a rejection of Task Dynamics or subsequent models inspired by it (e.g. Sorenson and Gafos, 2016; Tilsen, 2019), but a deepening of its predictive logic that is better able to predict major aspects of actual speech dynamics.

## Discussion and Conclusions

This paper contributes to the literature on motor control as a dynamical phenomenon, initiated by Bernstein (1967) and Feldman (1966), and extended to speech by Fowler et al. (1980), by showing how the low degree of freedom tasks of a motor control system are obtained *via* a dynamical computational process that starts out with a very large number of degrees of freedom (see also, Roon and Gafos, 2016; Tilsen, 2019). The contribution is to isolate the high degree system, the low degree system, and the extremely specific BVAM dynamical process as a candidate dynamical system that starts with the high degree of freedom system and ends with the low degree of freedom system. The evidence advanced in the last section is of the abstract and concrete types. A subtle abstract property of the phonological act of speech production is that gestures begin and end in time and are localized in space as *constrictions*. The simultaneous presence of Turing and Hopf bifurcations achieves the segmentation in space and time that we see in speech. In the Task Dynamic program, for instance, the tasks are almost all categorized in terms of constriction locations and degrees, as most phonological feature theories are structured into place and manner features. The current theory explains why the location/place and degree/manner distinctions are so prevalent. It is due to particulation. And two highly concrete properties of how constrictions actually form and relax, one qualitative (pivoting) and the other quantitative (proportional time to peak velocity of approximately 0.5) are reproduced by the simultaneous Turing-Hopf BVAM model. The only thing that had to be postulated is that the neural planning units affect the closure of the vocal tract at different points, but this is the usual assumption about what motoneurons do. The computational system presented answer the *how* question (material and formal cause), and not the *why* (final cause) of the reason for particulation. I believe that two of the main final causes for particulation are the ones discussed in the introduction: (1) to allow for many words built from a few basic particles (Abler, 1989; Studdert-Kennedy and Goldstein, 2003); (2) to allow the vocal tract to act as an acoustic signaling device (Stevens, 1972, 1989; Mrayati et al., 1988).

However, even though this model is hopeful in that it explains some subtle and other concrete properties of speech production, it is by itself not sufficient, and has fatal shortcomings as a comprehensive model. First, it needs to be shown that manipulation of the constants of the system can produce actual words of actual languages, which has not been done here. This is unlikely to be doable with this system, since as can be seen in Figure 7C, the particulation in both space and time is too periodic to be of use in describing actual words in actual languages. This model can almost be seen as a model of a *gagagaga* stage of CV babbling, and the search needs to continue for other reaction-diffusion systems with simultaneous Turing and Hopf instabilities or interactions between those two instabilities that take us beyond the *gagagaga* stage of CV babbling by adding *controlled* variation in constriction location and degree. In the field of phonetics this may seem to disqualify this model entirely, but work in mathematical physics for centuries has always sought to understand complex phenomena, many of which are far less complex than speech, by proposing models that explain simple abstract properties of the phenomenon first, and that is the approach taken here. Second, the model does not cease. It needs to become clear how the model can stop and produce a single word with just a few changes in constrictions in space and time. Therefore, part of the search for a refinement of the current model needs to take into consideration how the model can produce word length actions then stop, and start again. Third, some fundamental properties of speech having to do with prosody have not been mentioned, however there have been other dynamical approaches to prosody (Goldsmith and Larson, 1990; Prince, 1993; Goldsmith, 1994; Iskarous and Goldstein, 2018) that we believe can be combined with this model, since equations with Turing and Hopf pattern solutions have a multiscale structure (Kuramoto, 1975; Walgraef, 1997) that is actually quite similar to syllabification and metricity in speech as modeled by Goldsmith. Fourth, the actual spatial extent and temporal extent is known from many observational experiments, whereas the current model, using arbitrary units, does not generate these actually observed extents, however, rescaling of the variables is likely to allow the current model, or improvements, to match the macroscopic scales of speech. Fifth, even though there is plenty of evidence that neural computation is capable of generating Turing and Hopf patterns separately, the BVAM architecture used, has not, to my knowledge, been argued independently, to be a model of neural computations. One reaction-diffusion approach to cortical organization that has extensively used Turing, Hopf, and Turing-Hopf patterns as a foundation for brain macroscopic function has been presented in a series of papers by Steyn-Ross and her colleagues (Steyn-Ross and Steyn-Ross, 2013, 2017; Wang et al., 2014). In this work, the excitatory agent is the neurotransmitter Acetylcholin (ACh) and the inhibitory agent is GABA. Diffusion takes place through gap junctions, which communicate electrical signals between connected neurons. This group has specifically been quantitatively modeling global EEG waves that accompany the different stages of non-REM sleep. The authors show that measured signals of EEG are predicted based on simulation of models of neural interaction that are simplified by considering the input of each neuron to not be the specific other neurons that innervate it, but the mean of the entire network it is in. This *mean-field approximation* seems drastic, but is quite common in physics, and allows for prediction of actual solutions of a network as complex as a brain. Work by Cowan and his colleagues has tried to use more realistic approximations (Buice and Cowan, 2007). The work of Steyn-Ross specifically shows that a Turing-Hopf Bifurcation of the mean-field approximation plays a major role in the brain computations indicative of sleep. Therefore, even though we have not attempted to provide a brain-model that actually predicts the particulation we find in speech, there is some partial support for the possibility that Turing-Hopf patterns have a role to play in neural computation.

The nature of the microscopic units in this model is also uncertain. The conjecture we have offered so far is that the units are neural in nature. Another possibility is that the reaction diffusion equations to be sought are actually for the motions of tense hydrostatic muscle. Stoop et al.’s (2015) work on nonlinear elasticity theory has argued that geometrically and materially nonlinear material, the kind of material we know the tongue and other speech organs to be (Wilhelms-Tricarico, 1995), can yield reaction diffusion type equations of the Swift-Hohenberg type, and it is known that this type of equation has Turing and Hopf bifurcations (Cross and Hohenberg, 1993; Walgraef, 1997). Therefore, there could be a second conjecture that the equations sought are physical, not neural in nature. And even a third conjecture, the one we expect most likely to correspond to reality, where the equations are a combination of neural and muscular nature, non-dualistically connected.

## Data Availability Statement

The raw data supporting the conclusions of this manuscript will be made available by the authors, without undue reservation, to any qualified researcher.

## Author Contributions

The author confirms being the sole contributor of this work and has approved it for publication.

### Conflict of Interest

The author declares that the research was conducted in the absence of any commercial or financial relationships that could be construed as a potential conflict of interest.

## Footnotes

^1^Particulation is not negated by many types of complexity, such as the fact that certain contrasts have multiple temporal stages or multiple articulations (Goldsmith, 1976; Catford, 1977; Sagey, 1986; Browman and Goldstein, 1989; Steriade, 1994; Shih and Inkelas, 2019), since each of the stages and articulations of multiple articulations are themselves particulated. And it is also not negated by the fact that many gestures are highly dynamic or overlap, since even dynamic events in the vocal tract are delimited in time, and their changes occur at particular finite locations in the vocal tract (Iskarous, 2005; Iskarous et al., 2010); also, overlapped gestures are themselves particulated.

^2^There are tutorials on dynamical systems analysis intended for linguists in Sorenson and Gafos (2016) and Iskarous (2017). The meanings of equations presented here are described following the presentation of each equation; however, the reader interested in the details can consult these papers for background on dynamics.

^3^The discrepancy interpretation of the Laplacian ∇^2^ *A* is due to Maxwell (1871).

^4^I am excluding, here, traveling wave patterns, since these are not found in speech production (Iskarous, 2005), even though they may be involved in swallowing.
